# HEALTH: laparoscopic supracervical hysterectomy versus second-generation endometrial ablation for the treatment of heavy menstrual bleeding: study protocol for a randomised controlled trial

**DOI:** 10.1186/s13063-017-2374-9

**Published:** 2018-01-24

**Authors:** Kevin Cooper, Kirsty McCormack, Suzanne Breeman, Jessica Wood, Neil W. Scott, Justin Clark, Jed Hawe, Robert Hawthorn, Kevin Phillips, Angela Hyde, Alison McDonald, Mark Forrest, Samantha Wileman, Graham Scotland, John Norrie, Siladitya Bhattacharya, for the HEALTH Study Group, Siladitya Bhattacharya, Kevin Cooper, Kirsty McCormack, Neil Scott, Justin Clark, Jed Hawe, Robert Hawthorne, Kevin Phillips, Angela Hyde, Graham Scotland, John Norrie, Suzanne Breeman, Jessica Wood, Alison McDonald, Mark Forest, Samantha Wileman, Rebecca Bruce, Moira Richie, Fiona Cherry

**Affiliations:** 1 NHS Grampian, Aberdeen Royal Infirmary, Foresterhill Road, Aberdeen, AB25 2ZN UK; 2 Centre for Healthcare Randomised Trials, University of Aberdeen, Health Sciences Building, Foresterhill, Aberdeen, AB25 2ZD UK; 3 Medical Statistics Team, University of Aberdeen, Polwarth Building, Foresterhill, Aberdeen, AB25 2ZD UK; 4 Birmingham Women’s NHS Foundation Trust, Birmingham Women’s Hospital, Mindelsohn Way, Birmingham, B15 2TG UK; 5 Countess of Chester Hospital NHS Foundation Trust, Countess of Chester Health Park, Liverpool Road, Chester, CH2 1UL UK; 6 NHS Greater Glasgow and Clyde, Southern General Hospital, 1345 Govan Road, Glasgow, G51 4TF UK; 7 Hull and East Yorkshire Hospitals NHS Trust, Castle Hill Hospital, Castle Road, Cottingham, HU16 5JQ UK; 8 Royal College of Obstetricians and Gynaecologists Women’s Network, Regent’s Park, London, NW1 4RG UK; 9 Health Economics Research Unit, University of Aberdeen, Polwarth Building, Foresterhill, Aberdeen, AB25 2ZD UK; 10 Medical Statistics and Trial Methodology, Usher Institute of Population Health Sciences and Informatics, University of Edinburgh, Little France Road, Edinburgh, EH16 4UX UK; 11 Institute of Applied Health Sciences, University of Aberdeen, Polwarth Building, Foresterhill, Aberdeen, AB25 2ZD UK

**Keywords:** Heavy menstrual bleeding, Laparoscopic, Hysterectomy, Endometrial ablation techniques, Randomised controlled trial

## Abstract

**Background:**

Heavy menstrual bleeding (HMB) is a common problem affecting approximately 1.5 million women in England and Wales with a major impact on their physical, emotional, social and material quality of life. It is the fourth most common reason why women attend gynaecology outpatient clinics and accounts for one-fifth of all gynaecology outpatient referrals. Initial treatment in primary care is medical - either by means of oral or injected medication or the levonorgestrel-intrauterine system (Mirena®). If medical treatment fails then surgical treatment can be offered, either endometrial ablation (EA), which destroys the lining of the cavity of the uterus (endometrium), or hysterectomy, i.e. surgical removal of the uterus.

While effective, conventional hysterectomy is invasive and carries a risk of complications due to injury to other pelvic structures. The procedure can be simplified and complications minimised by undertaking a ‘supracervical’ hysterectomy where the cervix is left in situ and only the body of the uterus removed. Recent advances in endoscopic technologies have facilitated increased use of laparoscopic supracervical hysterectomy (LASH) which can be performed as a day-case procedure and is relatively easy for the surgeon to learn.

HEALTH (Hysterectomy or Endometrial AbLation Trial for Heavy menstrual bleeding) aims to address the question ‘Is LASH superior to second generation EA for the treatment of HMB in terms of clinical and cost effectiveness?’

**Methods/Design:**

Women aged < 50 years, with HMB, in whom medical treatment has failed and who are eligible for EA will be considered for trial entry. We aim to recruit women from approximately 30 active secondary care centres in the UK NHS who carry out both surgical procedures. All women who consent will complete a diary of pain symptoms from day 1 to day 14 after surgery, postal questionnaires at six weeks and six months after surgery and 15 months post randomisation. Healthcare utilisation questions will also be completed at the six-week, six-month and 15-month time-points.

**Discussion:**

Measuring the comparative effectiveness of LASH vs EA will provide the robust evidence required to determine whether the new technique should be adopted widely in the NHS.

**Trial registration:**

International Standard Randomised Controlled Trials, ISRCTN49013893. Registered on 28 January 2014.

**Electronic supplementary material:**

The online version of this article (10.1186/s13063-017-2374-9) contains supplementary material, which is available to authorized users.

## Background

Heavy menstrual bleeding (HMB) is a common problem which affects approximately 1.5 million women in England and Wales. For clinical purposes, HMB is defined as excessive menstrual blood loss which interferes with the woman’s physical, emotional, social and material quality of life (QoL), and which can occur alone or in combination with other symptoms [1].

The condition accounts for one-fifth of all gynaecology outpatient referrals and has a major impact on women’s physical, emotional, social and material QoL. The condition is initially treated by general practitioners, either by means of oral or injected medication or insertion of the levonorgestrel-intrauterine system (Mirena®). If medical treatment fails, surgical treatment can be offered, either in the form of endometrial ablation (EA), which destroys the lining of the cavity of the uterus (endometrium), or hysterectomy, i.e. surgical removal of the uterus. However, neither medical treatment nor EA can guarantee complete resolution of symptoms. Up to 59% of women on oral drugs [2] and 13.5% of those using the levonorgestrel-intrauterine system (Mirena) [3] require surgery within two years, while 19% of women treated by EA go on to have a hysterectomy for relief of their symptoms [4].

Hospital Episode Statistics data indicate that a total of 136,921 hysterectomies and 128,434 EAs for HMB were performed in England and Wales between April 1997 and December 2009 [5]. EA is commonly performed at present by means of second-generation or non-hysteroscopic procedures including thermal balloon EA and bipolar electrode EA (Novasure® (Hologic Inc.)).

The National Institute for Health and Care Excellence (NICE) guideline on HMB recommends both EA as well as hysterectomy as options for women with HMB resistant to medical treatment [1], but a significant minority of women treated with EA are likely need further EA or hysterectomy. A recent individual patient data meta-analysis [6] of results from randomised trials has shown that despite the greater invasiveness, longer hospital stay and prolonged recovery associated with conventional hysterectomy (removal of the uterus and the cervix), fewer women are dissatisfied with it in comparison with EA. Additionally, a cost-effectiveness model based on these data also favoured hysterectomy [7]. A Health Technology Assessment (HTA) evidence synthesis report [8] showed that one-quarter of all women who undergo EA will require subsequent gynaecological surgery, with just under one-fifth requiring hysterectomy. These findings, which are consistent with those of a relevant Cochrane review [9], suggest that the optimal surgical treatment for HMB unresponsive to medical treatment may well be hysterectomy, but its effectiveness needs to be balanced against its invasiveness and increased short-term and long-term morbidity [4].

Unlike conventional hysterectomy, the more recent approach of laparoscopic supracervical hysterectomy (LASH) removes the body of the uterus, which is responsible for menstrual bleeding, but conserves the cervix and the uterosacral ligament complex. It is minimally invasive, quick, relatively easy to learn and associated with low risk of complications, short hospital stay (under 24 h) and rapid recovery time [10, 11] and could potentially provide the benefits of a conventional hysterectomy without its morbidity and prolonged recovery time.

Before this technique is incorporated into routine clinical practice, it is important that it is subjected to robust evaluation. Authors of two small randomised trials comparing LASH with a first-generation EA—endometrial resection [11]—or second-generation EA—thermal balloon [10]—suggest that LASH could lead to a better QoL, but have emphasised the need for larger evaluative studies to confirm this, a view endorsed by the relevant Cochrane and HTA reviews.

The last decade has seen widespread use of laparoscopic techniques in gynaecology due to increased familiarity with the procedures, more sophisticated instruments, better training and greater surgical skill. As a result of this, LASH could be delivered by most general gynaecologists with minimal morbidity to women who are currently being treated with EA. Advances in perioperative care also means that, unlike conventional hysterectomy, hospital stay in women treated by this procedure may not be any longer than in those receiving EA.

HEALTH (Hysterectomy or Endometrial AbLation Trial for Heavy menstrual bleeding) is a multicentre randomised controlled trial (RCT) comparing LASH with second-generation EA (the current first line surgical treatment for HMB) in terms of clinical and cost-effectiveness. The trial is relevant and timely, as a robust evaluation of this new surgical option will provide much-needed high-quality evidence to underpin any decision to offer it as a preferred treatment.

## Methods/Design

### Study design

**Fig. 1:**
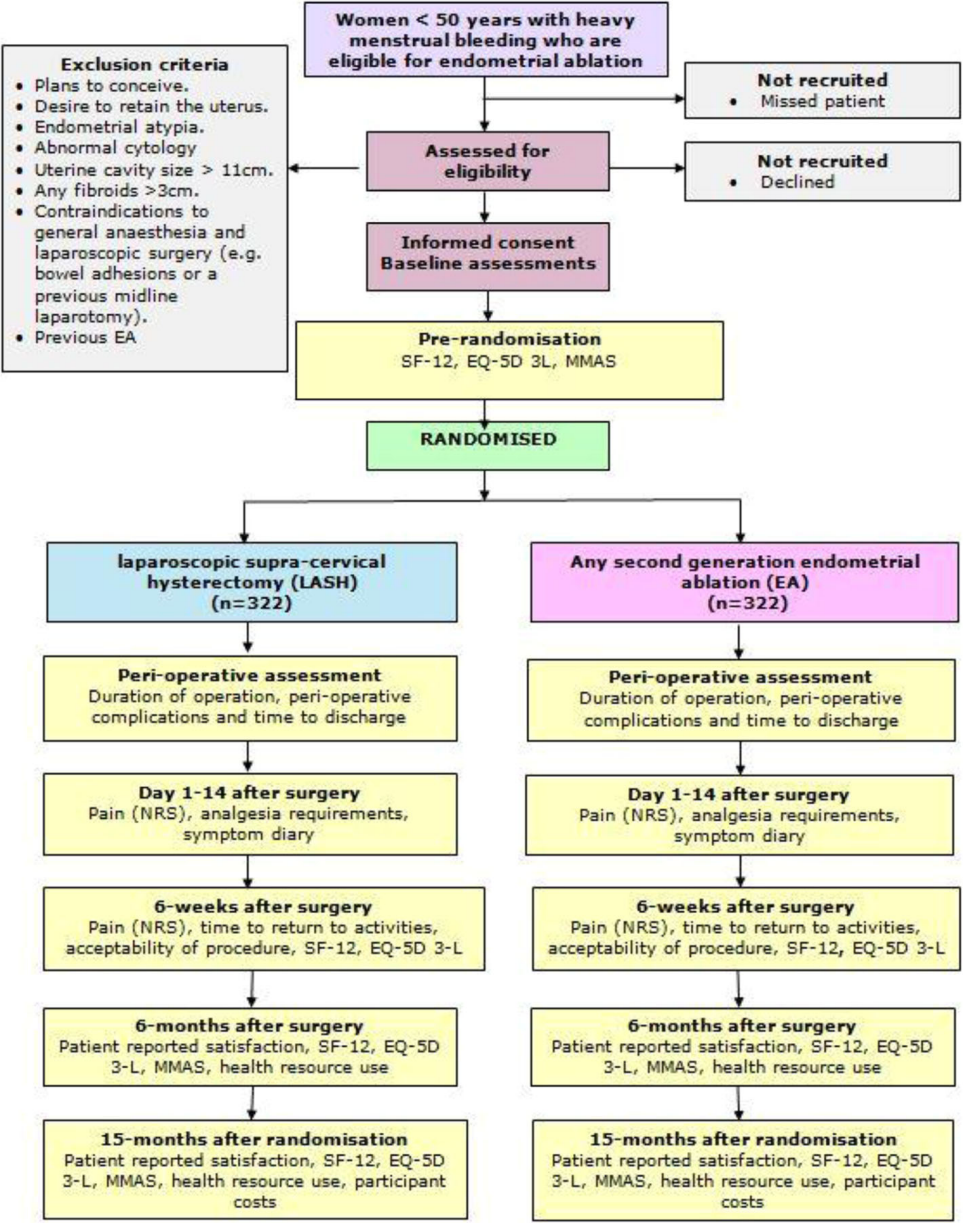
HEALTH flow diagram

We have designed a multicentre RCT of alternative surgical treatments for women with HMB. The trial structure is shown in Fig. 1 (flow diagram). The rationale for our proposed trial design reflects the uncertainties in the evidence base in this clinical area. EA is a successful treatment in the short term, but as around 20% of women who fail to benefit from this procedure will need further treatment such as repeat ablation or hysterectomy, it is important to address the impact of these events on relative cost-effectiveness. Conventional hysterectomy (where the cervix is removed along with the body of the uterus through an open procedure) is a definitive treatment, but is also potentially more morbid, with a longer postoperative recovery time. LASH offers the permanence of a conventional hysterectomy by means of a less invasive procedure with a quick recovery time.

### Study population

We aim to recruit women aged < 50 years with HMB who are eligible for EA from approximately 30 active secondary care NHS hospitals in the UK which can carry out both surgical procedures. Discussions at meetings facilitated by the relevant professional organisation, the British Gynaecological Endoscopy Society, and an online survey of members of this Society have confirmed that minimal access surgeons from these centres are willing to randomise women to either option.

### Inclusion and exclusion criteria

#### Inclusion criteria

1. Women aged < 50 years with HMB eligible for EA.
2. Women who are willing to be randomised between LASH and EA.

#### Exclusion criteria

1. Women with plans to conceive, endometrial atypia, abnormal cytology, uterine cavity size > 11 cm, any fibroids > 3 cm, contradictions for laparoscopic surgery (e.g. midline lower abdominal incision or known intrabdominal / pelvic adhesions) and previous EA.
2. Women who are unable to give informed consent or complete trial documentation.

### Trial interventions

This protocol addresses the comparison of two surgical operations for HMB: LASH and EA. The surgical procedures have been agreed and standardised by consensus within the research team and recruiting gynaecologists. EA will be performed using second-generation techniques under either local or general anaesthetic.

#### Laparoscopic supracervical hysterectomy (LASH)

LASH involves removal of the upper part of the uterus or the body by means of keyhole surgery facilitated by use of morcellation or culdotomy to remove the uterine corpus. The uterine body contains the endometrial cavity lined with tissue which undergoes cyclic growth and shedding each month thus causing menstrual bleeding. Increased access to specialised laparoscopic equipment and training means that LASH is quick and relatively easy to learn. It is associated with low morbidity, short hospital stay (under 24 h) and rapid recovery time. Unlike conventional total hysterectomy, the cervix is not removed, thus removing the need for bladder dissection, extended surgery around the cervix and often disruption of the uterosacral ligament complex. These extra steps, necessary for the removal of the cervix, can lead to serious complications such as injury to the bladder, ureters and blood vessels. As the cervix is retained, cervical smears are still required and although most women will cease to have periods after the procedure, light menstrual loss can occur in 5–10% of cases.

#### Endometrial ablation (EA)

EA aims to treat HMB by destroying the endometrium (lining of the womb) which is responsible for heavy periods. Historically, a number of methods have been used to achieve this. Initially, in operations involving so called ‘first-generation’ techniques, the interior of the uterine cavity was visualised endoscopically and the endometrial lining resected or ablated using electric diathermy or laser energy. More recently, ‘second-generation’ techniques which did not require hysteroscopic visualisation of the uterine cavity became popular. Current second-generation procedures used in the UK include thermal balloon EA and a bipolar radiofrequency electrode EA device known as Novasure® (Hologic, Inc.). Thermal balloon EA is undertaken by means of a silicone balloon which is introduced through the cervix into the uterine cavity. Hot fluid circulating within the balloon ensures endometrial destruction and the temperature and duration of treatment is carefully controlled electronically by means of a computer attached to the device. Novasure® uses radiofrequency energy delivered through an intrauterine mesh electrode which expands on insertion through the cervix to fit the shape of the uterine cavity. These EA techniques significantly reduce menstrual loss and result in complete cessation of bleeding in 40–50% of women [1]. Second-generation EA procedures can be performed either under general or local anaesthetic, costing the NHS £995 per procedure carried out as a day case in 2011/2012 [12].

### Identification and enrolment of potential participants

The consultant gynaecologist, dedicated research nurse or designated team member at outpatient gynaecology clinics and pre-assessment clinics in each recruiting centre will identify all eligible women referred from primary care for consideration of surgery for HMB. As local procedures at the participating hospitals are different, the timing and mode of approach to women and the consent process may vary in order to accommodate both the specific circumstances at each site and the needs of the women. The local recruitment team will give or send a patient information leaflet (PIL) describing the study to each eligible woman who will have the opportunity to discuss the study with her gynaecologist. Women will also have the opportunity to discuss all aspects of the proposed research with the local clinical team (staff at pre-admission clinics and ward staff while admitted), the Research Nurse, family and friends and, if appropriate, with their GP before admission. Women may decide to participate during an initial consultation with their gynaecologist, during a subsequent visit to hospital (e.g. a clinic appointment, a pre-assessment visit or when they are admitted for surgery) or alternatively at home. If the woman agrees to be contacted at home (recorded on the Surgical Assessment Form), she may receive a telephone call from the local research nurse to discuss any queries. Women who decide to participate following telephone counselling can either send their completed documents (consent form and baseline questionnaire) through the post to the local team at their treating hospital or bring it with them if they are returning to hospital for another consultation or surgery.

The PIL and consent form refer to the possibility of long-term follow-up to determine the incidence of future operations (Additional file 1).

### Randomisation and allocation

Eligible and consenting participants are randomised to one of the two study groups in a 1:1 allocation ratio using the randomisation application at the trial office at the Centre for Healthcare Randomised Trials (CHaRT). This randomisation application is available 24 h a day, seven days a week as both an interactive voice response (IVR) telephone system and as an Internet-based application. The randomisation will use a minimisation algorithm based on centre and age.

### Follow-up procedures

Eligible patients who have provided signed informed consent to participate in the study will complete the SF12, Menorrhagia Multi-Attribute Quality of Life Scale (MMAS) and EQ5D at baseline before being randomised to either LASH or EA. They will also complete a diary for days 1–14 post surgery, to record pain scores and the use of analgesics. At six weeks after surgery, participants will complete a questionnaire to measure Pain Numerical Rating Scales (NRS), time to return to normal activities and acceptability, EQ5D and SF12. At six months after surgery and at 15 months following randomisation, participants will complete the SF12, MMAS, EQ5D, satisfaction with treatment and questions about healthcare utilisation. Participants will receive up to two reminders by post, email or phone, taking into account any preferences they may have for mode of communication.

### Change of status/withdrawal procedures

Participants will remain in the trial unless they chose to withdraw consent or if they are unable to continue for a clinical reason. If a participant withdraws consent, we will not send them questionnaires but will seek permission for the research team to continue to collect outcome data from their healthcare records (via the case report forms [CRFs]). All other changes in status—with the exception of formal withdrawal of consent—will mean the participant is still followed up for all study outcomes wherever possible.

### Safety

The HEALTH trial involves procedures for the surgical management of HMB in women which are well established in clinical practice. Adverse effects may occur during or after any type of surgery. In this trial, the following events are potentially expected: admission to high dependency unit/intensive care unit; emergency hysterectomy; laparotomy; port site hernia; blood transfusion; wound infection; lower urinary tract infection; endometritis; blood-stained vaginal discharge; anaesthetic complications; low grade pyrexia; blood loss; haematoma; constipation; pelvic discomfort/pain; internal bleeding or injury; pulmonary embolism (PE); deep vein thrombosis (DVT); injury to the wall of the uterus; bladder injury; bowel injury; ureteric injury; and voiding dysfunction.

### Outcome measures

This RCT will assess and compare LASH with standard technique of EA in respect of: condition-specific QoL; patient-reported satisfaction; and other patient-reported outcomes (complications, recovery details, further gynaecological surgery and modelled long-term cost-effectiveness).

#### Primary outcome measure

The co-primary (clinical) outcomes will be: (1) MMAS, a condition-specific QoL outcome [13] in the range of 0–100 based upon six domains, measured at 15 months after randomisation; and (2) patient satisfaction, measured on a 6-point scale (from ‘totally satisfied’ to ‘totally dissatisfied’) measured at 15 months after randomisation. Mean (SD) MMAS scores will be reported provided the data are normally distributed. Satisfaction will be treated as a binary variable: ‘totally satisfied’ vs other responses. We will address these two co-primary outcomes in a hierarchical fashion. First, we will consider the patient satisfaction, and if this shows a statistically significant difference (*p* < 0.05), then we will consider the disease-specific QoL MMAS outcome. Both will need to achieve statistical significance at *p* < 0.05 for the null hypothesis to be rejected. By specifying this hierarchy, we do not need to apply any adjustment for multiple comparisons, since the overall false-positive error is controlled at an alpha of 0.05. Together these measures are comprehensive, intuitive and accepted by patients and the clinical community and have been used in previous trials and studies by the Aberdeen group and others in the field [14–16].

The primary economic outcome is the incremental cost (to the health service) per quality-adjusted life year (QALY) gained (LASH vs EA). We will calculate this from within-trial health service costs (resource use collected via CRFs and patient questionnaires, and valued using standard unit prices) and generic QALYs (derived from responses to the EQ-5D). We will derive the incremental cost per QALY gained for LASH vs EA from generalised linear regression models adjusting for baseline health status and other important covariates.

#### Secondary outcome measures

Patient-reported: MMAS score at six months after surgery; patient-reported satisfaction at six months after surgery; acceptability of procedure measured at six weeks after surgery; severity of postoperative pain using a pain NRS measured at 1–14 days and six weeks after surgery; symptom diary days 1–14 after surgery (including analgesic use); generic health-related QoL (SF-12, EQ-5D 3-L) measured at six months after surgery and at 15 months after randomisation. We will describe outcomes using mean (SD) or frequency (%) as appropriate.

Clinical: duration of operation; perioperative complications and recovery details including analgesia requirements; time to discharge; further gynaecological surgery by 15 months after randomisation. We will describe outcomes using mean (SD) or frequency (%) as appropriate.

Economic: wider societal costs associated with changes in productivity based on information on the time taken to return to normal activities (following intervention) combined with questions on work productivity delivered during the follow-up period. Furthermore, we will develop a simple Markov model, based on within trial data supplemented by available published data on the requirement for further gynaecological surgery over time (following the alternative procedures) and use it to extrapolate cost-effectiveness beyond 15 months after randomisation.

While the analyses within this application are based upon an initial 15 months after randomisation follow-up, we also anticipate collecting long-term information on further gynaecological surgery by utilising Hospital Episode Statistics for England and Wales and Information Services Division (ISD) data for Scotland. We will use these data in the future to revise the extrapolated longer-term estimates of cost-effectiveness for LASH vs EA.

### Schedule of data collection

The components of follow-up are shown in Table 1.

**Table 1:** HEALTH measurement of outcomes: components and timing

	Pre randomisation		Post surgery			Post randomisation
	Baseline	Surgery	Days 1–14	6 weeks	6 months	15 months
Baseline CRF	X					
Surgical details		X				
Pain NRS symptom diary			X			
Pain NRS				X		
Time to return to normal activities				X		
Acceptability				X		
Satisfaction					X	X
MMAS,	X				X	X
EQ-5D 3-L, SF-12	X			X	X	X
Healthcare utilisation					X	X
Participant costs						X

## Sample size, proposed recruitment rate and milestones

### Sample size

The specification of the target difference was driven by two criteria: (1) what target difference would be important if it existed; and (2) what would be a realistic difference [17] given the interventions under evaluation. With regards to (2), the observed rates in the recent IPD meta-analysis of abdominal hysterectomy vs first-generation EA [6] would lead to a target difference of odds ratio (OR) of 2.84 (95% vs 87%) for patient satisfaction. Such an OR also equates to a medium-sized standardised effect (Cohen’s d). This requires 292 participants per group for a two-sided test with 90% power. This size would also be more than sufficient to allow a small- to medium-sized (0.3, Cohen’s d) standardised effect in the co-primary outcome, MMAS, to be detected; this is a target difference for MMAS that can be viewed as important and has observed in other areas for similar outcomes. This would equate to being able to detect a target difference of 10 points on the 0–100 scale, given a standard deviation of 33 points or less. Given these assumptions for the co-primary outcomes, and additionally allowing for 10% missing data, 648 participants in total are required.

### Recruitment rates and milestones

The original recruitment projection was based on 30 active centres participating, with the expectation that they would contribute a minimum of 26 women per centre over 21 months of recruitment (months 6–26 inclusive). We expected a staggered recruitment of centres with all centres active by the end of month 12. Recruitment at all sites was projected to be 50% of the projected monthly total in the first month and reduced recruitment in the peak holiday months of August and December.

### Revised recruitment rates and milestones

At steady state, the recruitment rate was assumed to be approximately 62 women per month, although recruitment was actually slower than anticipated. This occurred for a number of reasons, principally patient preference, lack of equipoise among clinical colleagues and organisational issues at the recruiting centres.

The revised projections for the extension period are based on a conservative estimate of the recruitment trend observed over a six-month period from September 2015 to February 2016 inclusive, resulting in an expected recruitment rate of 25 participants per month. As a result, a 12-month extension to the recruitment phase is necessary to achieve the original target sample size (648 participants in total).

**Fig. 2:**
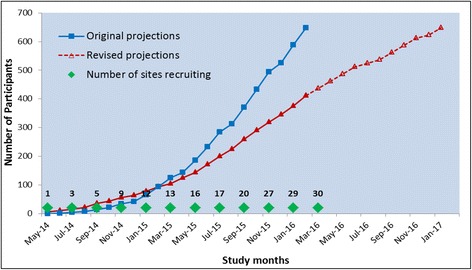
Recruitment projections

**Fig. 3:**
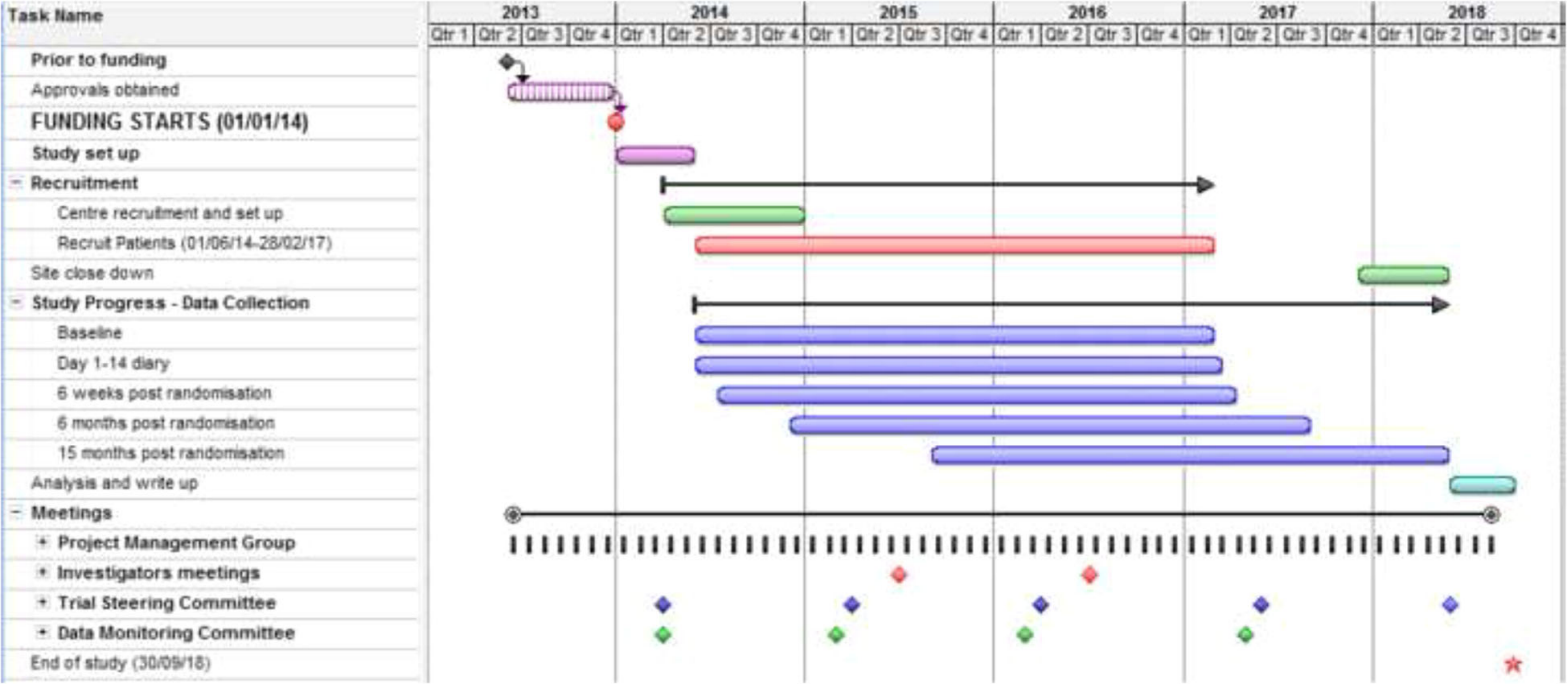
HEALTH Gantt

The original and revised recruitment projections can be found in Fig. 2 and the revised project timetable and milestones are shown in the Gantt chart in Fig. 3.

### Statistical analysis

We will base all analyses on the intention-to-treat principle, analysing women in the groups to which they are randomised. We will conduct all study analyses according to a statistical analysis plan agreed in advance by the Trial Steering Committee. We will conduct the analyses at two-sided 5% significance level with corresponding 95% confidence interval generated as appropriate. Full details may be found in the separate statistical analysis plan.

We will conduct the analysis of the two co-primary outcomes (patient satisfaction and MMAS) independently. We will analyse patient satisfaction (‘totally satisfied’ vs others) using a logistic regression model with adjustment for minimisation factors. We will use a complete-case analysis for the main analyses, with no imputation for missing data. Sensitivity analyses will assess the impact of varying the dichotomisation cut-off and adjusting for clustering at centre and surgeon levels. Sensitivity analyses (such as using a multiple imputation approach) will also explore what influence missing data might have on the robustness of our findings and where feasible modelling non-ignorable (informative) missing data mechanisms. A further analysis of patient satisfaction will use a proportional odds model utilising the underlying ordinal (Likert) scale (Ologit function, Statacorp, 2012). We will analyse the MMAS using linear regression adjusted for baseline and minimisation factors or an ordinal model if the data are found to be skewed. We will analyse secondary outcomes using generalised linear models adjusted for minimisation factors (and, when appropriate, a baseline measure).

### Planned subgroup analyses

We will perform exploratory subgroup analyses for the following groups: uterine cavity length (8 cm ≤ vs > 8 cm); menstrual pain (dysmenorrhoea) at baseline (‘severe’ vs non-‘severe’ – determined using a 5-point Likert scale); fibroids (present or absent); patient age < 40 or > 40 years. We will conduct the pre-specified subgroup analyses by including the corresponding treatment by subgroup interaction term in the corresponding regression models for the co-primary outcomes (patient satisfaction and MMAS). No other subgroup analyses are planned. We will state the subgroup analyses as exploratory and evaluate at the 5% two-sided significance level.

### Proposed frequency of analyses

We will perform a single statistical analysis when 12-month follow-up data have been collected. An independent Data Monitoring Committee will review confidential interim analyses of accumulating data at its discretion but at least annually.

## Economic evaluation

The economic analysis will consist of a trial based analysis of individual patient level cost and effect (QALY) data and a decision modelling component to inform cost-effectiveness in the longer term.

For the within-trial analysis, we will estimate total costs to the health service, wider costs to society associated with lost productivity and QALYs for each individual patient enrolled in the RCT. We will estimate costs of the initial intervention procedures from resource use data recorded on the CRFs of each individual patient (including time in theatre, staff present, any perioperative complications and length of stay in hospital post treatment) coupled with routine unit cost data [12, 18]. We will also value any subsequent contacts with primary and secondary care (collected from patient questionnaires at six months after surgery and 15 months after randomisation), for each patient using nationally accepted sources of unit costs. Since the EQ-5D is the recommended instrument for deriving QALY weights by NICE (https://www.nice.org.uk/process/pmg9/chapter/foreword), we will use participant responses to this instrument (at baseline, six weeks and six months after surgery and 15 months after randomisation) to derive QALYs. The SF-12 is being included as another potentially more sensitive measure of general health-related QoL and will provide an alternative means for estimating QALYs via the SF-6D scoring algorithm. We will undertake this as a sensitivity analysis at 12 months. Productivity losses will be estimated based on the reported time taken to return to normal activities (assessed at six weeks after surgery) and responses to work productivity questions at six months after surgery and 15 months after randomisation. We will value time lost from paid employment using national age-/sex-specific average gross wage rates [19]. We will estimate the value of time lost from alternative non-paid activities using appropriate shadow prices.

Analysis of the patient level cost and QALY data will use appropriately specified generalised linear regression models adjusted for baseline EQ-5D score and minimisation factors applied during randomisation [20]. From these analysis models, the co-efficient for the treatment allocation group will provide estimates of the incremental costs and QALYs associated with LASH vs EA. We will characterise uncertainty surrounding the joint estimates of incremental costs and effects by running the regression models on a large number of bootstrapped samples obtained, with replacement, from the original trial sample. This process will generate a large number of estimates of the incremental costs and effects, capturing any correlation between them. We will plot these results on the incremental cost-effectiveness plane and use them to derive a cost-effectiveness acceptability curve, indicating the probability of LASH being cost-effective (at 12 months) given different notional values of decision-makers’ willingness to pay per QALY gained. The primary analysis will assess cost-effectiveness from the health service perspective, but we will also conduct a secondary analysis incorporating wider costs to society. As a further step to help present the 12-month findings in a meaningful way for decision-makers, we will present all costs and outcomes within a cost–consequence balance sheet. This will summarise all the costs and trial outcomes by treatment allocation group and indicate which treatment group each outcome favours.

While the within-trial analysis will be useful for informing cost-effectiveness in the short term, previous research suggests that a longer time horizon may be required to determine the relative cost-effectiveness of LASH vs EA [8], as a result of EA being less costly and effective in the short term but associated with higher failure rates and subsequent surgery beyond 12 months. Therefore, we will develop a simple Markov model to simulate the recurrence of symptoms and need for subsequent treatment over time, in order to estimate cost-effectiveness in the longer term. We will construct the model in consultation with clinicians and based on a review of existing decision models developed in the field. Input parameters will initially be informed by the within-trial analysis (to determine initial treatment costs and outcomes and the probability of any subsequent treatment events/complications occurring within 12 months). This will be supplemented with published data on recurrence and the need for further gynaecological surgery (repeat EA, LASH or conventional total hysterectomy) following EA and LASH. The model will incorporate the initial health service costs of treatment, ongoing costs associated with successful and unsuccessful treatment, and costs associated with subsequent surgery. We will apply utility weights (obtained from the trial data) to the alternative states in the model, allowing modelled QALYs to be estimated. We will run the model over a five-year period (the time point by which most women would be expected to have completed any subsequent required treatment), though we will also explore the impact of adopting longer time horizons. Linkage of participants’ records to health episode statistics will allow future quantification of the incidence of repeat gynaecological surgery, providing a means for validating/updating initial model based predictions.

We will carry out probabilistic and deterministic sensitivity analysis to characterise the uncertainty surrounding the model based estimates of incremental costs and effects of LASH vs EA. For the Probabilistic Sensitivity Analysis (PSA), we will assign an appropriate distribution to each model input parameter (reflecting the degree of uncertainty surrounding it due to sampling variation) and we will analyse the model a large number of times, each time randomly drawing a value for each input parameter from its assigned distribution [21]. This process will generate a large number of estimates of the incremental costs and effects. We will use cost-effectiveness acceptability curves to summarise the findings from the PSA. Further deterministic analysis will assess the sensitivity of the model based estimates to further choices over sources of parameter estimates and any structural assumptions required when constructing the model.

## Research governance, data protection and sponsorship

### Research governance

The trial is under the auspices of CHaRT based at the Health Services Research Unit (HSRU), University of Aberdeen. This will ensure compliance with Research Governance and provide centralised trial administration, database support, and economic and statistical analyses. CHaRT is a registered Clinical Trials Unit with particular expertise in running multicentre RCTs of complex and surgical interventions.

The two Aberdeen-based co-Chief Investigators will ensure, through the Trial Steering Committee (TSC), that adequate systems are in place for monitoring the quality of the study (compliance with the principles of Good Clinical Practice) and appropriate expedited and routine reports, to a level appropriate to the risk assessment of the study.

### Data protection

We will ensure that data collected during the course of the research is handled confidentially and accessed only by members of the trial team. We will store participants’ details on a secure database under the guidelines of the 1988 Data Protection Act and regular checks and monitoring are in place to ensure compliance. Data are stored securely in accordance with the Act and archived to a secure data storage facility. The senior IT manager (in collaboration with the Chief Investigator) will manage access rights to the dataset. We will allocate participants an individual specific trial number and we will anonymise their details on the secure database. We anticipate that anonymised trial data may be shared with other researchers to enable international prospective meta-analyses. To comply with the fifth Principle of the Data Protection Act 1998, personal data will not be kept for longer than is required for the purpose for which it has been acquired.

#### Data handling, record keeping and archiving

The local investigator and/or research nurse working in each hospital site will enter clinical data into the database, together with data from questionnaires completed at clinic. Staff in the trial office will enter data collected on questionnaires returned by post to the trial office. Staff in the trial office will also work closely with local research nurses to ensure that the data are as complete and accurate as possible. Extensive range and consistency checks will further enhance the quality of the data.

## Discussion

The HEALTH trial is the largest ever randomised controlled trial of alternative surgical treatments for women with HMB eligible for EA. It is designed to test the hypothesis that LASH is superior to second-generation EA in terms of patient satisfaction, QoL and costs.

Since the trial was first designed there have been several modifications to the protocol, none of which would have had a significant impact on the trial design and conduct. The key modifications include a change to the timing at which the final questionnaire (which captures the primary outcome) is triggered (12 months post surgery to 15 months post randomisation) and changes to the exclusion criteria where ‘previous endometrial ablation’ and ‘abnormal cytology’ were added and ‘submucous fibroids distorting the uterine cavity’ was changed to ‘any fibroids > 3 cm distorting the uterine cavity’. However, both these changes were made early in the trial.

The key practical challenges of this trial have been participant recruitment and retention. Early in the recruitment period, we noted issues with patient preference, which was heightened by the fact that LASH was being offered in some centres as part of the normal care pathway. Several strategies have been adopted to address these issues which have included increased engagement with the participating centres (regular updates, newsletters, research nurse teleconferences), reiterating the importance of the clinical question including explaining to practitioners that LASH is not endorsed by current NICE guidelines and opening further centres using specific criteria to identify those with a strong interest in the clinical question.

Strategies adopted to ensure response rates to the postal questionnaires remained within expected levels included a newsletter which pre-notified the participants of the questionnaire delivery (primary outcome only), the option to complete the questionnaire online and monetary incentives (unconditional £25 gift voucher for UK high street stores). The full impact of the strategies to improve recruitment and retention will be discussed in the full trial report.

## Trial status

The first participant was randomised into the trial on 21st May 2014 and the trial is currently open to recruitment in 33 UK centres, with the last participant follow-up expected in May 2018.

HEALTH protocol Version 1.9: 1st November 2016

## End notes

### Safety

The HEALTH trial involves procedures for the surgical management of HMB in women which are well established in clinical practice. Adverse effects may occur during or after any type of surgery.

### Definitions

An adverse event (AE) is any untoward medical event affecting a clinical trial participant. Each initial AE will be considered for severity, causality or expectedness and may be reclassified as a serious event or reaction based on prevailing circumstances.

A serious adverse event (SAE), is any AE, that:

- results in death;
- is life-threatening (i.e. the individual was at risk of death at the time of the event; it does not refer to an event which hypothetically might have caused death if it were more severe);
- requires hospitalisation or prolongation of existing hospitalisation;
- results in persistent or significant disability or incapacity;
- is otherwise considered medically significant by the investigator.

*Note: Hospitalisations for treatment planned before randomisation and hospitalisation for elective treatment of a pre-existing condition will not be considered as an AE. Complications occurring during such hospitalisation will be AEs or SAEs as appropriate.*

### HEALTH-specific expected adverse events

In this trial the following AEs are potentially expected: admission to HTU/ITU; emergency hysterectomy; laparotomy; port site hernia; blood transfusion; wound infection; lower urinary tract infection; endometritis; blood-stained vaginal discharge; anaesthetic complications; low grade pyrexia; blood loss; haematoma; constipation; pelvic discomfort/pain; internal bleeding or injury; PE; DVT; injury to the wall of the uterus; bladder injury; bowel injury; ureteric injury; and voiding dysfunction.

### Procedures for detecting, recording, evaluating and reporting AEs and SAEs

#### Detecting AEs and SAEs

Non-serious events will be recorded in the CRFs. Planned primary care or hospital visits for conditions other than those associated with HMB or consequence of surgery will not be collected or reported. Hospital visits (planned or unplanned) associated with further interventions due to HMB (e.g. further surgery) will be recorded as an outcome measure, but will not be reported as SAEs.

Any SAEs related to the participants’ HMB treatment that are not further interventions (e.g. if a participant is admitted to hospital for treatment of infection) will be recorded on the SAE form. In addition, all deaths for any cause (related or otherwise) will be recorded on the SAE form.

Within HEALTH, ‘relatedness’ is defined as an event that occurs as a result of a procedure required by the protocol, whether or not it is either: (1) the specific intervention under investigation; or (2) it is administered outside the study as part of normal care.

#### Recording AEs and SAEs

Depending on severity, when an AE/SAE occurs, it is the responsibility of the Investigator (or delegate) to review appropriate documentation (e.g. hospital notes, laboratory and diagnostic reports) related to the event. The Investigator (or delegate) should then record all relevant information in the CRF and on the SAE form.

Information to be collected includes dose, type of event, onset date, Investigator assessment of severity and causality, date of resolution as well as treatment required, investigations needed and outcome.

#### Evaluating AEs and SAEs

All AEs will be assessed in respect of seriousness, relationship to study intervention, whether expected or unexpected and, therefore, whether constituting a SAE by the local PI, CI or their deputies.

#### Assessment of seriousness

The Investigator should make an assessment of seriousness as defined above in ‘Definitions’.

#### Assessment of causality

The Investigator must make an assessment of whether the AE/SAE is likely to be related to any of the research procedures according to the following definitions:

- Related: resulted from administration of any of the research procedures;
- Unrelated: where an event is not considered to be related to any of the research procedures.

Alternative causes such as natural history of the underlying disease, concomitant therapy, other risk factors and the temporal relationship of the event to the treatment should be considered.

#### Assessment of expectedness

When assessing expectedness refer to the expected events.

#### Reporting AEs and SAEs

##### Reporting responsibilities of the CI

When an SAE form is uploaded onto the trial website, the Trial Manager will be automatically notified. If, in the opinion of the local PI and the CI, the event is confirmed as being *serious* and *related* and *unexpected*, the CI or Trial Manager will notify the sponsor within 24 h of receiving the signed SAE notification. The sponsor will provide an assessment of the SAE.

The CI (or Trial Manager) will report any related and unexpected SAEs to the main REC within 15 days of the CI becoming aware of it. All related SAEs will be summarised and reported to the Ethics Committee, the Funder and the Trial Steering Committee in their regular progress reports.

If all the required information is not available at the time of reporting, the Investigator must ensure that any missing information is provided as soon as this becomes available. It should be indicated on the report that this information is follow-up information of a previously reported event.

## Additional files

Additional file 1: Consent form. (DOCX 167 kb)

Additional file 2: Authorship publication. (DOCX 12 kb)

Additional file 3: Authorship policy. (DOCX 15 kb)

Additional file 4: A completed SPIRIT checklist. (PDF 114 kb)

## Abbreviations

CHaRT: Centre for Healthcare Randomised Trials
CI: Chief Investigator
CRF: Case report form
DMC: Data monitoring committee
EA: Endometrial ablation
EQ-5D: EuroQol Group’s 5 dimension health status questionnaire
GP: General practitioner
HEALTH: Hysterectomy or Endometrial AbLation Trial for Heavy menstrual bleeding
HMB: Heavy menstrual bleeding
HSRU: Health Services Research Unit
HTA: Health Technology Assessment
ISD: Information Statistics Division
ISRCTN: International standard randomised controlled trial number
IVR: Interactive voice response (randomisation)
LASH: Laparoscopic supracervical hysterectomy
MMAS: Menorrhagia Mulit-Attribute QoL Scale
NHS: National Health Service
NICE: National Institute for Health and Care Excellence
NIHR: National Institute Health Research
NRS: Numerical Rating Scale
PI: Principal Investigator
PIL: Patient information leaflet
PMG: Project management group
PSA: Probabilistic sensitivity analysis
QALY: Quality-adjusted life year
QoL: Quality of life
RCT: Randomised controlled trial
TSC: Trial steering committee
UK: United Kingdom
