# Supplementary material for: HEALTH: laparoscopic supracervical hysterectomy versus second-generation endometrial ablation for the treatment of heavy menstrual bleeding: study protocol for a randomised controlled trial
Source: Trials. 2018 Jan 24;19:63. doi: 10.1186/s13063-017-2374-9 (PMC5784594; doi:10.1186/s13063-017-2374-9)
Supplement: Supplementary file 1 — Consent form. (DOCX 167 kb) [file 13063_2017_2374_MOESM1_ESM.docx]

| Participant Study No | | | | | |
| --- | --- | --- | --- | --- | --- |
|  |  |  |  |  |  |
|  |  |  |  |  |  |
|  |  |  |  |  |  |

**HEALTH TRIAL**

**Consent Form**

**
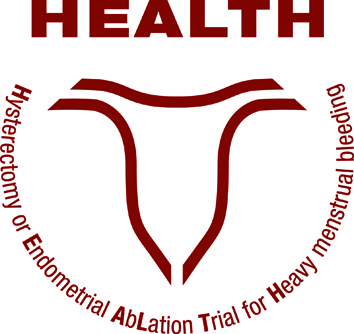
**

|  | |  | | | |
| --- | --- | --- | --- | --- | --- |
| **By initialling each box and signing this form:**  **1) I agree that I have** | | **Please initial**  **ALL boxes** | | | |
| been given the Information Sheet about the study (Version number 2.4, date 18/12/2015). I have had the opportunity to consider the information, ask questions and have had these answered satisfactorily. I understand the purpose of the study and I know what my involvement will be | | |  |  | |
|  |  |  |  |  | |
|  |  | |  |  |  |
| **I understand that** | | | | | |
| 1. my participation is voluntary and that I am free to withdraw at any time without giving any reason, without my medical care or legal rights being affected | | |  |  | |
|  |  | |  |  |  |
| 1. information relevant to the HEALTH Study may be collected from my hospital and NHS records, including Office of National Statistics (ONS) and NHS central registers | | |  |  | |
|  | | |  |  |  |
| 1. relevant sections of my medical notes and data collected during the study may be looked at by individuals directly involved in the trial, from the University of Aberdeen or from the NHS Boards or Trusts, where it is relevant to my taking part in this research. I give permission for these individuals to have access to them. | | |  |  |  |
|  |  |  |  |  | |

| 1. my personal contact details will be kept confidentially and securely by the study office in Aberdeen. I agree that the study co-ordinators can use my contact details to send me study questionnaires and to contact me by phone, post or email. |  |  |  |
| --- | --- | --- | --- |
|  |  |  | |
|  |  |  |  |

| 1. my family doctor (GP) will be told that I am taking part in this study | | | | |  |  |
| --- | --- | --- | --- | --- | --- | --- |
|  | | | | |  |  |
| 1. I may be contacted in the future for 5-year follow-up of this study | | | | |  |  |
|  | | | | |  |  |
| **I agree to take part in the study** | | | | |  |  |
|  | | | |  | | |
| Your signature (participant) | | | |  | | |
|  |  | | | |  | |
| Your name in block capitals | | | |  | | |
|  |  | | | |  | |
| Date | |  | | | | |
|  |  | | | |  | |
| **To be completed by the local team member taking consent** | | | | | | |
| I confirm that I have explained to the person named above, the nature and purpose of the study and the procedures involved | | | | | | |
| Signature | | |  | | | |
|  | | |  | | | |
| Name in block capitals | | |  | | | |
|  | | |  | | | |
| Date | |  | | | | |
|  |  | | | | | |

HEALTH Study Office, Centre for Healthcare Randomised Trials (CHaRT), Health Services Research Unit,

University of Aberdeen, Scotland AB25 2ZD; Tel 01224 438405; Fax 01224 438165; email health@abdn.ac.uk

Copies: Original for trial office in Aberdeen (white copy); 1 for participant (yellow copy);

1 to be kept in medical notes (pink copy); 1 for research site file (blue copy).
