# Supplementary material for: HEALTH: laparoscopic supracervical hysterectomy versus second-generation endometrial ablation for the treatment of heavy menstrual bleeding: study protocol for a randomised controlled trial
Source: Trials. 2018 Jan 24;19:63. doi: 10.1186/s13063-017-2374-9 (PMC5784594; doi:10.1186/s13063-017-2374-9)
Supplement: Supplementary file 2 — Authorship publication. (DOCX 12 kb) [file 13063_2017_2374_MOESM2_ESM.docx]

**AUTHORSHIP PUBLICATION**

All RCTs conducted by CHaRT have a commitment to publish the findings of the research. At a minimum this trial will have a results paper published in a peer- reviewed medical/scientific journal. If all grant-holders and researcher staff fulfil authorship rules, group authorship will be used under the collective title of ‘the HEALTH Trial Group’. If one or more individuals have made a significant contribution above and beyond other group members but where all group members fulfil authorship rules, authorship will be attributed to the named individual(s) and the HEALTH Trial Group.

For reports which specifically arise from the trial but where all members do not fulfil authorship rules (for example, specialist sub-study publications), authorship should be attributed to the named individual(s) for the HEALTH Trial Group.

To safeguard the integrity of the main trial, reports of explanatory or satellite studies will not be submitted for publication without prior arrangement from the Project Management Group.

We intend to maintain interest in the study by publication of HEALTH newsletters at intervals for staff and collaborators. Once the main report has been published, a lay summary of the findings will be sent in a final HEALTH Newsletter to all involved in the trial.

Further details on the publication policy can be found in Additional file 2.
